# Genotypic Variation in Maize Root Hydrotropism and Its Association with Shoot Growth and Water Use Efficiency Under Partial Root–Zone Drying

**DOI:** 10.3390/plants15101571

**Published:** 2026-05-21

**Authors:** Yuxin Guan, Zhihua Zhong, Jiaxin Zhao, Danning Li, Yibo Liu, Zichen Ma, Muyu Gu, Xueqin Han, Yafang Wang

**Affiliations:** 1 College of Grassland Agriculture, Northwest A&F University, Yangling 712100, China; 18835249548@163.com (Y.G.); zhihua@nwafu.edu.cn (Z.Z.); zhaojiaxin@nwafu.edu.cn (J.Z.); lidanning@nwafu.edu.cn (D.L.); liu3024978598@163.com (Y.L.); 15093835989@163.com (Z.M.); 2 Department of Biology and Microbiology, South Dakota State University, Brookings, SD 57007, USA; muyu.gu@jacks.sdstate.edu; 3 Institute of Tropical Eco-Agricultural Science, Yunnan Academy of Agricultural Sciences, Yuanmou 651300, China

**Keywords:** maize, hydrotropism, fixed partial root–zone drying, water use efficiency, drought stress

## Abstract

Drought severely limits maize yields. Water–saving irrigation methods like partial root–zone drying (PRD) can improve water use efficiency (WUE) but often result in variable yield responses among genotypes. We hypothesized that differences in root hydrotropism might contribute to some of this variability. Seven maize varieties were evaluated for hydrotropic response in a controlled moisture–gradient assay and then grown for five weeks under fixed PRD versus full irrigation in a greenhouse. The different maize varieties exhibited distinct hydrotropic behaviors: roots of V6 and V7 bent toward water much faster and more strongly, while V2 responded slowly with minimal curvature. Under PRD, genotypes also differed in root distribution and shoot performance. However, hydrotropism alone did not guarantee good shoot maintenance. One strongly hydrotropic genotype (V7) still suffered a large biomass reduction under PRD. Overall, genotypes that maintained better shoot water status, along with larger stem diameter and higher shoot water content, achieved the highest WUE under PRD. These results indicate that root hydrotropism varies widely in maize varieties. This variation was associated with shoot traits and WUE under PRD, suggesting that the benefit of hydrotropism for drought adaptation may depend on complementary shoot characteristics. Breeding for drought–resilient maize may therefore require combining strong root hydrotropism with the ability to maintain shoot function under water deficit.

## 1. Introduction

Drought stress is a major factor limiting maize productivity worldwide, as water deficits during the growing season sharply reduce growth and yield [1,2]. Agriculture accounts for roughly two–thirds of global freshwater use. Therefore, improving crop water use efficiency (WUE) is critical for sustainable maize production under increasing water scarcity [3]. One irrigation strategy to conserve water is partial root–zone drying (PRD), in which water is applied to only part of the root zone while the remainder is left dry [4]. In practice, PRD can be implemented by alternately watering halves of the root system or by maintaining a fixed wet–dry split [5]. This approach has been shown to reduce irrigation requirements and enhance WUE in many crops [4,6]. The physiological basis is that roots in the drying soil send abscisic acid and other signals to partially close stomata and slow water use, while roots in the moist soil continue to supply water to the plant [7]. However, yield responses to PRD can be variable. Some genotypes maintain near–normal growth, whereas others suffer significant yield penalties despite the water savings [8]. For example, maize genotypes that sustain greater lateral root foraging into the moist portion of the soil can better preserve yield under PRD conditions. These observations suggest that inherent root system response traits might modulate the success of PRD in different varieties.

Roots can dynamically adjust their growth orientation in response to environmental cues, including moisture gradients [9]. Root hydrotropism is the directional growth of roots toward areas of higher water potential, which is critical for plants to survive under water–limited conditions [10]. In contrast to gravitropism, hydrotropism allows roots to bias their development laterally or obliquely toward wetter soil zones, thereby potentially improving water acquisition under heterogeneous moisture conditions [11]. Mechanistically, hydrotropic curvature is thought to involve specialized sensing in the root cap and differential hormone signaling, such as that of auxin and abscisic acid, which inhibits growth on the side of the root closer to water [12,13,14]. Furthermore, light, reactive oxygen species (ROS), and Ca^2+^ are also thought to mediate the root hydrotropic response, but their detailed molecular mechanisms have not yet elucidated [15]. The identification of *Arabidopsis* mutants with impaired hydrotropic responses (e.g., *nhr1*, *ahr1*, and *miz1*) has significantly deepened our knowledge of the molecular mechanisms governing root hydrotropism [16]. Although hydrotropism has been well documented in laboratory systems, its variation among crop cultivars and its contribution to drought adaptation in agronomic contexts remain relatively unexplored.

Previous studies have shown that maize plants displaying a robust hydrotropic response grow better under drought and partial lateral irrigation [17]. In this study, we hypothesized that maize varieties might differ in their degree of root hydrotropic responsiveness, and that this trait could influence their performance under PRD irrigation. In a fixed PRD scenario, where one part of the root zone is consistently dry, a strongly hydrotropic genotype might concentrate more roots in the irrigated zone. This would support better shoot water status and productivity. Conversely, a weakly hydrotropic genotype might not reallocate roots as effectively, potentially experiencing greater water stress. To test these hypotheses, we first quantified genotypic differences in root hydrotropism among seven maize varieties (V1–V7) using a controlled hydrotropism assay. We then grew the same varieties under fixed PRD versus full irrigation (well–watered control) in a greenhouse and compared their root system partitioning, shoot growth maintenance, and WUE. Specifically, our aims were (i) to measure how quickly and strongly each variety’s roots bend toward a lateral water source; and (ii) to determine whether varieties differing in hydrotropic response also show distinct root allocation patterns, shoot biomass retention, and WUE under a lateral soil moisture gradient. By linking these two experiments, we seek to assess the potential role of hydrotropism in maize drought tolerance and to identify traits that contribute to high WUE with minimal yield penalty under PRD.

## 2. Results

### 2.1. Genotypic Differences in Root Hydrotropism

**Figure 1:**
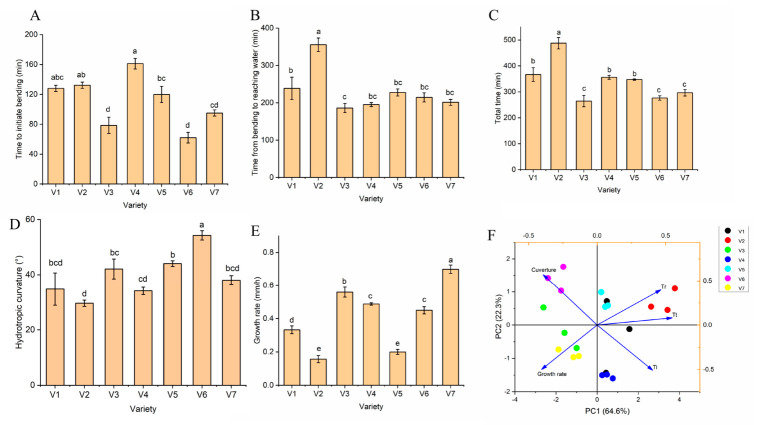
Hydrotropic responses of different maize varieties. (**A**) Time to initiate positive bending toward the wet pad; (**B**) time to touch the wet pad after initial bending; (**C**) total bending time from initial bending to reaching the water source; (**D**) root curvature after completion of hydrotropic bending; (**E**) root elongation rate during the hydrotropic response; (**F**) principal component analysis (PCA) of the parameters shown in (**A**–**E**) for all varieties. Different lowercase letters above the columns indicate significant differences among varieties (*p* < 0.05).

A unique hydrotropism analyzer was used to evaluate the hydrotropic response of different maize varieties. This system allowed us to assess hydrotropism in terms of hydrotropic response time, root growth rate, and hydrotropic curvature. The seven maize varieties showed clear differences in root hydrotropic response in the controlled assay. In general, varieties V3, V6, and V7 initiated hydrotropic root bending sooner than V1, V2, and V4. These same three varieties (V3, V6, and V7) also completed the bending response more rapidly, reaching the wet pad in less total time (Figure 1A–C). By contrast, V2 exhibited the slowest response, with a longer initiation bending time and a longer total time to reach the water. Final bending angles (hydrotropic curvature) also varied: V6, V5, and V3 achieved greater curvature toward the water source, whereas V2 ended with the smallest curvature (Figure 1D). Root elongation rates during the tropic response differed as well, with V7 and V3 showing the highest growth rates and V2 and V5 the lowest (Figure 1E). A principal component analysis (PCA) of the hydrotropism traits further summarized these differences (Figure 1F). The PCA separated the varieties along two major axes. The first principal component (PC1) was dominated by the time–based parameters (initiation bending time and total time), and the second component (PC2) was dominated by curvature and growth rate. Consistent with the univariate results, V3, V6, and V7 clustered together in PCA space, characterized by faster and stronger hydrotropic responses (short bending times and high curvature/growth). In contrast, V2 was separated due to its much slower and weaker response.

### 2.2. Plant Performance Under Fixed Partial Root–Zone Drying (FPRD)

Plant growth parameters and WUE were significantly affected by irrigation regime, maize genotype, and their interaction (Table 1). Irrigation regime significantly influenced all measured traits, including root dry weight, root water content, shoot dry weight, shoot water content, stem diameter, plant height, and WUE. In contrast, genotype had a significant effect on most traits, with the exception of root water content, shoot dry weight, and plant height. Significant irrigation × genotype interactions were also observed for root dry weight, shoot water content, stem diameter, and WUE.

#### 2.2.1. Root Growth Under Fixed Partial Root–Zone Drying (FPRD)

**Figure 2:**
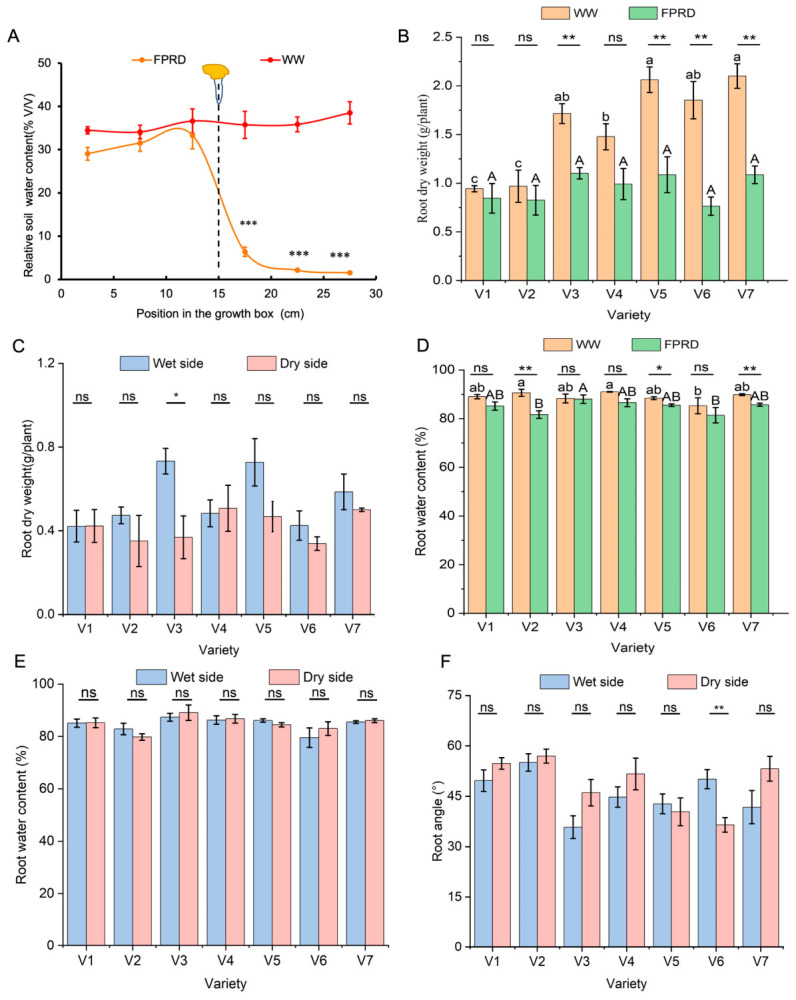
Soil water content and root growth responses under fixed partial root–zone drying (FPRD) and well–watered (WW) conditions in different maize varieties. (**A**) Soil water content at six horizontal positions in the growth chamber after 5 weeks of FPRD (left: wet side; middle: where the maize seedlings were planted; right: dry side) and WW treatment. *** indicates a significant difference in soil water content between WW and FPRD conditions at the same position (*p* < 0.001). (**B**) root dry weight of WW and FPRD plants; (**C**) root dry weight on the wet side and dry side of FPRD plants; (**D**) root water content of WW and FPRD plants; (**E**) root water content on the wet side and dry side of FPRD plants; (**F**) root angle on the wet side and dry side of FPRD plants. Different lowercase and uppercase letters above the bars indicate significant differences (*p* < 0.05) among varieties under WW and FPRD, separately, for Figure 2B,D. *, **, and ns indicate differences between WW and FPRD conditions within the same maize genotype at *p* < 0.05, *p* < 0.01, and not significant, respectively.

In the greenhouse trial, soil moisture measurements at harvest were even under the WW (well–watered) conditions. In contrast, a pronounced lateral moisture gradient occurred in the FPRD chambers, with significantly lower water content on the dry side (Figure 2A). Under WW conditions, V5 and V7 exhibited higher root dry weight than V4, while V1 and V2 had the lowest root dry weight. However, root dry weight was comparable among the seven varieties under FPRD. Accordingly, a significant decrease in root dry weight was observed for V3, V5, V6, and V7 under FPRD compared to WW conditions, whereas the differences for V1, V2, and V4 were not significant (Figure 2B). These results suggest that FPRD significantly inhibited root growth in V3, V5, V6, and V7. Regarding root biomass distribution between the two sides, V3 was the only variety that showed higher root dry weight on the wet side than on the dry side (Figure 2C). However, V4 was the only variety that showed a numerical increase in root dry weight on the dry side compared with the wet side, but the difference was not statistically significant. FPRD also affected root water content. Under WW conditions, V2 and V4 maintained higher root water content than V6, whereas under FPRD conditions, V3 exhibited higher root water content than V2 and V6. Root water content significantly decreased for V2, V5, and V7 under FPRD compared to WW conditions (Figure 2D). However, root water content was comparable between the wet side and the dry side (Figure 2E). Overall, FPRD significantly reduced both root dry weight and water content in V5 and V7, suggesting that their roots were sensitive to the soil water gradient. Root architecture was also influenced by the wet–dry divide: the angle of root growth differed between the wet–side and dry–side roots in V6 (Figure 2F). The data indicated that the seven maize varieties differed in the extent and direction of root system adjustment under a lateral moisture gradient. V6 preferentially allocated roots to the wet region, whereas other varieties exhibited a more generalized inhibition of rooting under drought. 

#### 2.2.2. Shoot Performance and WUE Under FPRD

**Figure 3:**
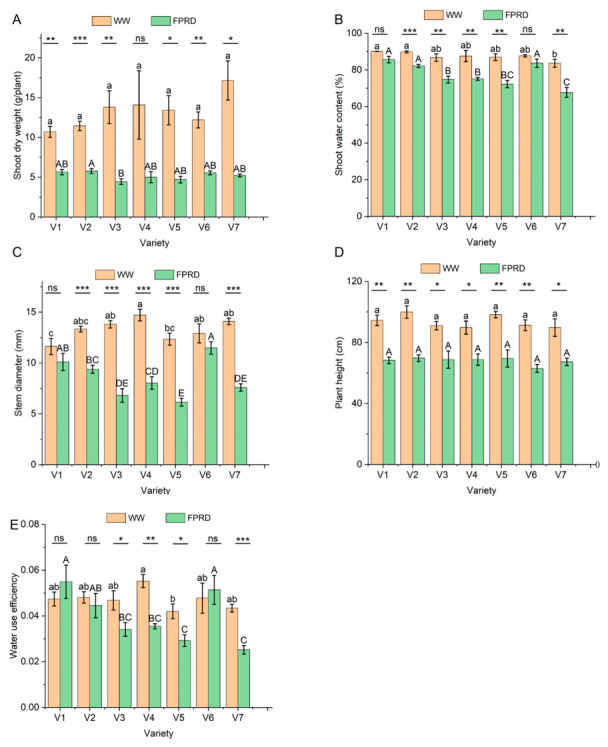
Shoot growth and water use efficiency (WUE) under fixed partial root–zone drying (FPRD) and well–watered (WW) conditions in different maize varieties. (**A**) Shoot dry weight; (**B**) shoot water content; (**C**) stem diameter; (**D**) plant height; (**E**) WUE. Different lowercase and uppercase letters above the bars indicate significant differences among varieties under WW and FPRD, respectively (*p* < 0.05). *, **, and *** indicates differences between WW and FPRD conditions within the same maize genotype at *p* < 0.05, *p* < 0.01, and *p* < 0.001, respectively; ns indicates not significant.

FPRD had significant impacts on shoot growth, and these effects differed widely among the maize varieties. Under WW conditions, shoot dry weight was comparable among the seven varieties. However, FPRD significantly reduced shoot biomass for all varieties except V4 (Figure 3A). Under FPRD, shoot dry weight was higher in V2 than in V3. Varieties V1 and V2 maintained higher shoot water content than V7 under both WW and FPRD conditions, suggesting that V1 and V2 possess a strong water retention capacity. Notably, for V1, shoot water content under FPRD was comparable to that under WW conditions (Figure 3B). Stem diameter was greater in V4 than in V5 and V1 under WW. Under FPRD, V6 exhibited a larger stem diameter than all other varieties except V1. Accordingly, all varieties except V1 and V6 showed a significant decrease in stem diameter under the FPRD conditions (Figure 3C). Although FPRD significantly reduced plant height, plant height remained comparable among the maize varieties under both WW and FPRD conditions (Figure 3D). WUE refers to the ratio of water used in metabolism to water lost through transpiration and other processes. Under WW conditions, WUE differed among varieties, with V4 exhibiting higher WUE than V5 (Figure 3E). Under FPRD, V1, V2, and V6 showed higher WUE than V5 and V7, resulting in comparable WUE under WW and FPRD for these three varieties. In contrast, WUE greatly decreased for V3, V5, and V7 under FPRD.

### 2.3. Trait Associations Under WW and FPRD Conditions

**Figure 4:**
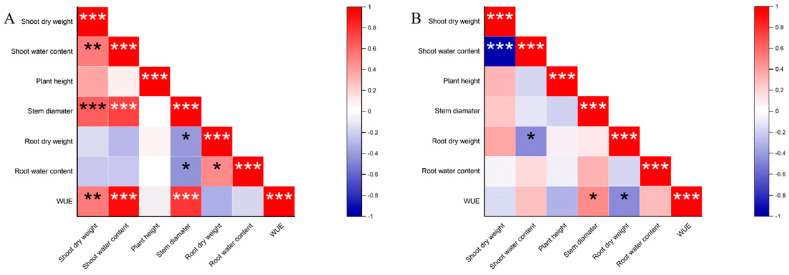
Pearson’s correlation matrices of shoot and root traits under well–watered (WW) and FPRD conditions. The traits included are shoot dry weight, shoot water content, plant height, stem diameter, root dry weight, root water content, and WUE. (**A**) Pearson’s correlation matrix for trait data under well–watered (WW) conditions; (**B**) Pearson’s correlation matrix under fixed partial root–zone drying (FPRD) conditions. Color intensity reflects the strength of positive (red) or negative (blue) correlations. *, **, and *** indicate significant correlations at *p* < 0.05, *p* < 0.01, and *p* < 0.001, respectively.

The correlations among morphological and physiological traits shifted notably between full irrigation (WW) and FPRD conditions. Under WW conditions, WUE showed a strong positive correlation with shoot growth traits. In particular, Pearson’s correlation analysis revealed that WUE was positively correlated with shoot dry weight and, especially, with stem diameter (Figure 4A). This indicates that, when water was not limiting, larger plants with greater stem diameter tended to use water more efficiently. Under FPRD conditions, however, the pattern of trait correlations changed (Figure 4B). WUE remained positively correlated with stem diameter, but WUE became negatively correlated with root dry weight under FPRD. In other words, under the FPRD regime, genotypes with larger total root mass tended to have lower WUE, and vice versa. Additionally, under FPRD, shoot water content was negatively correlated with both shoot dry weight and root dry weight (Figure 4B). This suggests that plants maintaining higher tissue water content under FPRD were not necessarily the largest in dry matter. In fact, those with very high shoot or root biomass tended to have somewhat lower relative water content, potentially reflecting a growth–versus–hydration trade–off in the FPRD environment.

**Figure 5:**
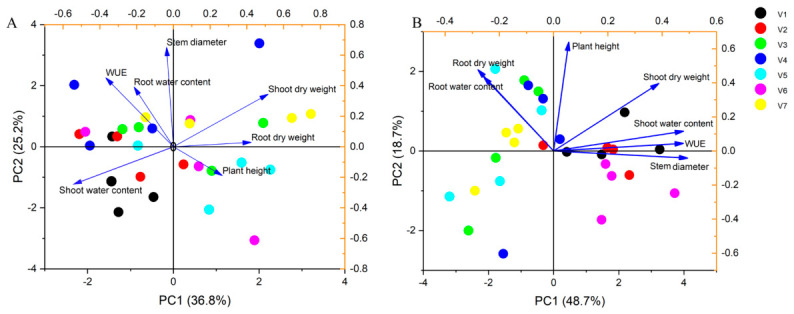
Principal component analysis (PCA) of shoot and root traits under well–watered (WW) and FPRD conditions. (**A**) PCA of trait profiles for the seven varieties under WW conditions; (**B**) PCA of trait profiles under FPRD conditions.

The PCA results provided a complementary perspective on how trait relationships and varietal differences depended on watering regime. A PCA of the seven varieties based on their shoot and root traits showed limited separation among genotypes under WW conditions (Figure 5A). Under WW, the first two principal components did not clearly cluster the varieties into any obvious group; most genotypes overlapped in trait space, indicating similar trait profiles when water was ample. In contrast, under FPRD conditions, the PCA revealed distinct separation for certain varieties (Figure 5B). Notably, V1, V2, and V6 separated from the others along PCA axes that were associated with greater stem diameter, higher shoot water content, and higher WUE. Meanwhile, varieties like V3, V5, and V7 clustered differently, being associated with traits such as higher root biomass or reduced shoot metrics under FPRD. This separation implies that FPRD imposed new trait trade–offs that distinguished the genotypes. Collectively, the correlation and PCAs highlight stem diameter as a consistently important trait: maintaining a larger stem diameter was strongly linked to improved WUE under the stress of lateral drying. In summary, hydrotropic root growth was one adaptation observed. Hoverve, the genotypes that most successfully coped with FPRD were those that sustained their shoot water status and structural growth (e.g., stem diameter), thereby achieving a favorable balance between water use and biomass production.

## 3. Discussion

### 3.1. Genotypic Variation in Root Hydrotropism in Maize

The phylogenetic analysis based on a 5K chip revealed substantial genetic variation among the seven commercial maize hybrids. This study demonstrated that these hybrids exhibited significant genotypic variation in root hydrotropism. Using a controlled hydrotropism assay, we found that some maize varieties initiated and completed curvature toward a water source much faster than others. For instance, V3, V6, and V7 showed a rapid hydrotropic response, whereas V2 responded much more slowly and achieved a smaller overall curvature. In this study, we focused on the typical commercial forage maize hybrids grown in China, as these hybrids are mainly cultivated in arid and semi–arid areas. Previous research analyzed the hydrotropic response in primary roots of 47 maize elite DTMA (Drought Tolerant Maize for Africa) hybrids and identified three robust and three weak hybrids, respectively [17]. Sáenz–Rodríguez et al. further assayed maize hydrotropic curvature of 72 DTMA hybrid lines and identified robust and weak lines [18]. These studies indicate that maize genotypes showed great variation in hydrotropic response. The present results extend this knowledge to different commercial maize hybrids. The mechanisms underlying the observed variation were not investigated here, but differences in root cap sensing or hormonal sensitivity could be involved [19]. Our previous study showed that plant hormones, such as auxin, ABA, and zeation, were involved in the maize hydrotropic response [13]. On the other hand, varieties with slower or weaker hydrotropism may prioritize gravitropic growth [20]. The practical implication is that hydrotropism, as a root behavioral trait, is not uniform across maize germplasm. This raises the question of whether breeding for improved hydrotropic response could be beneficial for drought adaptation. Notably, the range of hydrotropic behaviors we observed provides a basis for selecting contrasting genotypes for further study further in more complex soil environments.

### 3.2. Root System Adjustments Under FPRD

Under FPRD treatment, root system responses differed markedly among the maize varieties, and these differences were only partly explained by hydrotropism. We hypothesized that a strongly hydrotropic variety would concentrate more roots on the irrigated side when one side of its root system was drying, thereby accessing water to support the plant. Indeed, one of the fast–responding hydrotropic genotypes (V3) displayed this expected behavior. It allocated significantly greater root biomass to the wet side than to the dry side, consistent with root growth toward available water. Such a pattern aligns with the idea that roots can sense favorable moisture in part of the soil and preferentially proliferate there [21]. In agricultural contexts, this could be advantageous by allowing the plant to use the irrigated zone of soil more efficiently under FPRD. However, not all genotypes followed this pattern. Surprisingly, V7, which had one of the strongest hydrotropic curvatures in the assay, did not show an obvious wet–side preference in terms of final root biomass. Instead, V7’s total root growth was substantially inhibited under FPRD on both sides. This suggests that although V7’s roots are capable of bending toward water, other factors, such as the stress from the dry side or inherent growth habits, limited its overall root proliferation in the wet zone [22]. Consequently, V7 did not effectively capitalize on the available wet soil, despite its intrinsic hydrotropic tendency.

The varying root angles provide additional insight. V6, another fast–responding line, maintained a wider root spread on the wet side under FPRD, implying that it was exploring that moist region, whereas its dry–side roots grew more narrowly, which was perhaps due to suppression by drought [23]. These qualitative differences underscore that root system architecture under heterogeneous moisture is a complex trait, influenced by both tropisms and growth capacity [24,25].

From an agronomic perspective, the ability to maintain root function in at least part of the soil under drought is critical [26]. Our results indicate that some maize varieties (V3) can adjust their root distribution to partially compensate for a drying zone, whereas others (V7) suffered a more uniform decline in root growth. Interestingly, V1, which had only moderate hydrotropism in the assay, exhibited only a minor reduction in total root mass under FPRD. This indicates that V1 maintained overall root growth across both wet and dry sectors, despite its moderate hydrotropism. In contrast, V6 and V7 both showed notable reductions in total root biomass under FPRD, suggesting a drought–sensitive root growth response in those genotypes [26]. These findings highlight that strong hydrotropism does not necessarily guarantee robust root performance under field–like drought conditions. A genotype must also sustain root growth capacity under stress. Variability in root drought tolerance, xylem sap abscisic acid levels, or carbon allocation to roots under stress could all contribute to why some varieties’ root systems cope better with FPRD than others [27,28,29]. Overall, breeding for drought resilience may require combining traits that promote root redistribution (such as hydrotropism) with traits that ensure root persistence in drying soil.

### 3.3. Shoot Maintenance, WUE, and the Hydrotropism Paradox Under FPRD

One of the key results of our greenhouse experiment was that the variety rankings for shoot performance under FPRD did not align neatly with the rankings for hydrotropic root response. We initially expected that a variety with rapid, aggressive hydrotropism would experience less shoot water deficit and thus maintain higher biomass under FPRD. However, our data showed a more complex situation and did not exactly match this expectation. V7, despite being highly hydrotropic, had the poorest shoot performance under FPRD, with its shoot dry weight decreased by 70%, and its WUE declined substantially. Meanwhile, V1, which responded comparatively slowly in the hydrotropism assay, was the top performer for shoot maintenance, retaining 53% of its biomass and even comparable WUE under FPRD and WW. V6 aligned with the expectation: it had both strong hydrotropism, minimal losses in shoot water content, larger stem diameter, and a higher WUE. These contrasting cases suggest that hydrotropism is beneficial only in the context of other supporting traits. If a plant’s shoots cannot tolerate even mild water stress, or if its overall root growth is severely stunted by drought, then simply bending roots toward water may not rescue its productivity.

Our results point to shoot water status and stem diameter as key traits for great performance under FPRD. Stem diameter was a consistently important trait: varieties that maintained a larger stem diameter (V1 and V6) also maintained higher shoot water content and achieved better WUE. In this context, stem diameter served as a practical index for water status or turgor, because retention of stem diameter growth under drought indicates better hydration and reduced shrinkage [30]. PRD is known to invoke chemical signals, such as ABA from dry roots, which partially close stomata [31]. Therefore, more work is needed to examine the physiological response mechanisms, such as ABA content and stomatal conductance. V7 showed stronger growth suppression, whereas V1 and V6 maintained growth under FPRD. As a result, V1 and V6 used the limited water more efficiently. They continued growing, albeit slowly, and ended up producing relatively more biomass per unit water, resulting in greater WUE under FPRD than under WW. In contrast, V7’s aggressive response might conserve water to a great extent, but it also drastically limits photosynthesis and growth, yielding poor WUE. This is consistent with reports that PRD effectiveness depends on balancing water saving with acceptable yield loss [8]. Excessive growth suppression in response to drying soil can negate the benefits.

The correlation between root biomass and WUE under PRD was significant and negative. This suggests that the heavy root investment under FPRD did not translate into improved shoot performance. Previous studies showed a carbon–allocation trade–off in which sustained underground allocation under drought occurs at the expense of shoot growth, lowering WUE [32]. Meanwhile, a drought–tolerant plant may restrain root growth and allocate more resources to the shoot, resulting in a higher shoot–to–root biomass ratio under drought stress [33]. In our experiment, V1 maintained shoot vigor despite a modest root system, yielding more biomass per unit water. Nevertheless, extremely reduced rooting (as in V7) was clearly detrimental. An intermediate strategy that maintaining sufficient roots for uptake on the wet side while avoiding excessive belowground allocation may be optimal under fixed PRD.

In summary, the interplay between root behavior and shoot response under FPRD is complex. A strong hydrotropic response can be part of a drought adaptation strategy, but it is insufficient on its own. Our results suggest that maize varieties achieving the best performance under FPRD were those that integrated decent root hydrotropism with resilient shoot traits. The best performers combined effective root placement with resilient shoot traits (V6), whereas V7 expressed only the former and V1 primarily the latter. This insight is important for crop improvement. Selection solely for enhanced root tropisms might not yield drought–tolerant cultivars unless accompanied by traits such as drought–resistant shoots or balanced root growth regulation. Traits such as stem diameter maintenance, as well as higher shoot water content, could serve as useful indicators of a genotype’s ability to endure PRD stress [34].

In conclusion, this work highlights that maize varieties can differ widely in root tropic responses and in how they partition growth between roots and shoots under uneven water supply. Breeding programs aiming to improve drought tolerance should therefore consider multiple traits, examining both below–ground for efficient water foraging and above–ground for sustained growth under stress. Hydrotropism is an appealing trait to enhance water uptake from moist soil pockets, but its value will be realized only in genotypes that can also withstand the accompanying drought signals. Our study provides a framework and baseline data for selecting and combining such traits. As water becomes an increasingly limiting resource, approaches like PRD will be valuable tools, and understanding plant responses to these regimes will help in tailoring crop varieties for maximum benefit with minimum trade–offs.

## 4. Materials and Methods

### 4.1. Plant Materials

Seeds of seven maize (*Zea mays* L.) varieties were obtained from Beijing Dajingjiu Agricultural Development Co., Ltd., Beijing, China: Zhengsiyu 2 (V1), Zhongyu 335 (V2), Yuqingzhi 23 (V3), Yayuqingzhu 8 (V4), Jingkeqingzhu 932 (V5), Heyu 36 (V6), and Dajingjiu 26 (V7). These are representative of the typical commercial hybrids grown in China. The detailed pedigree information for these commercial hybrids is provided in Table S1. To characterize the genetic diversity among the seven maize hybrids, we performed genotyping using a maize 5K SNP chip by Chengdu Tiancheng Future Technology Co., Ltd. (Chengdu, China). The resulting genotypic data confirmed substantial genetic variation among the hybrids. This diversity provides a useful basis for exploring associations between genotypic differences and physiological traits.

### 4.2. Hydrotropism Assay

The hydrotropism assay system used in this study was modified based on the Arabidopsis root hydrotropism system described by Takahashi N et al. [35] and modified for maize primary roots following Wang et al. [13]. The hydrotropic analyzer consisted of a polystyrene foam container (30 × 20 × 25 cm^3^, length × width × height). On one side of the container, a wet triple–layer cotton cloth was positioned to provide a water–saturated source. On the opposite side, a plastic tray (20 × 14 × 5 cm^3^, length × width × height) containing 200 mL of saturated potassium carbonate (K_2_CO_3_) solution was placed to maintain a low water potential environment. A stable moisture gradient was established between the water–saturated cotton strip and the saturated K_2_CO_3_ solution to induce root hydrotropic response. Maize seeds were germinated vertically between two layers of moistened paper towel at 24 °C in darkness until primary roots reached 1.2–1.5 cm in length. The kernel was wrapped in moist cotton and placed vertically in the hydrotropic analyzer with the root tip 2 mm away from the wet cloth (water source). Time–lapse images were collected using a video camera (HC–V770K, Panasonic Corporation, Osaka, Japan) to quantify four hydrotropic response traits: (i) the time to initiate bending, (ii) the time to reach the water source after bending (total bending time), (iii) the final bending curvature, and (iv) the root elongation rate during the bending response. Root bending angle was measured from images captured at the end of hydrotropic bending using IC Measure software (version 2.0.0.286, the Imaging Source Europe GmbH, Bremen, Germany). A straight line was drawn from the root tip to the base of the root (the midpoint where curvature began). The angle between this line and the original root axis (vertical direction) was measured as the root bending angle. Three biological replicates per variety were tested, with experiments carried out in multiple independent runs.

### 4.3. Greenhouse FPRD Experiment

Experiments were conducted in a greenhouse at Northwest A&F University (Yangling, China). The greenhouse experiment was arranged in a split–plot design with four biological replicates. Irrigation regime of FPRD and WW was assigned to main plots within each block. Within each main plot, the seven maize hybrids were randomly assigned to sub–plots. Acrylic growth chambers (30 × 5.5 × 30 cm^3^, length × width × height) were filled with equal masses of a loam–based potting mix (Pindstrup Mosebrug A/S, Ryomgaard, Denmark). One pregerminated seedling (with approximately 1.3 cm primary root) was transplanted into the center of each chamber (one maize plant for each chamber). The top of each chamber was covered with aluminum foil to minimize direct evaporation from the soil surface. All plants were watered uniformly and grown under well–watered conditions until they reached the three–leaf stage.

At the three–leaf stage, two irrigation regimes were imposed for a treatment period of five weeks. In the WW conditions, water was applied to the soil on both sides of the chamber to replace 100% of the estimated evapotranspiration. In the FPRD treatment, water was applied to only one side of the root system (designated the ‘wet side’) at 50% of evapotranspiration demand, while no water was added to the opposite side (the ‘dry side’). Thus, FPRD maintained a consistent lateral soil moisture contrast, with one side irrigated at half the rate of a fully watered control and the other side left dry. Water was delivered by pipette every three days for precise control. The volume of water added to each chamber was adjusted based on cumulative transpiration (measured by weighing chambers) and recorded to calculate total water consumption per plant.

During the experiment, the greenhouse temperature ranged from 30 ± 5 °C to 20 ± 5 °C during day and night. After five weeks of treatment, all plants were harvested for root and shoot measurements. The relative soil water content at different positions in the chamber was measured using a soil moisture analyzer (SM150 Soil Moisture Kit, Delta–T Devices Ltd., Cambridge, UK).

#### 4.3.1. Root Measurements

At harvest, roots were carefully removed and separated by chamber side. For FPRD plants, roots from the wet side and dry side were kept separate to obtain side–specific measurements. For WW plants, the root system was similarly divided into two halves corresponding to the two sides of the chamber for comparison. Roots were gently rinsed free of soil and blotted dry. The fresh weight of the root sample from each side was recorded immediately after excision. Roots were then oven–dried (first at 105 °C for 10 min, then at 70–80 °C until reaching constant weight) and weighed to determine dry weight. Root water content was calculated as the difference between fresh weight and dry weight on a fresh weight basis for each sample. Total root dry weight per plant and total root water content per plant were also computed by combining the two sides. The root angle of wet and dry side was measured using a digital angle finder (Deli Group Co., Ningbo, China).

#### 4.3.2. Shoot Measurements

Before harvest, plant height was measured with a tape measure (from the base of the stem to the tallest leaf tip), and stem diameter was measured at the first node using a vernier caliper. After harvest, shoots were separated from roots and immediately weighed to obtain shoot fresh weight. Shoots were dried using the same protocol as for roots (105 °C for 10 min, then 70–80 °C to constant mass) to determine shoot dry weight. Shoot water content was calculated as shoot fresh weight minus dry weight on a fresh weight basis.

#### 4.3.3. Water Use Efficiency (WUE)

WUE at the chamber scale was defined as the shoot dry weight produced per total water consumed over the treatment period. For each plant, cumulative water consumption was calculated as the total volume of water added to the chamber during the five weeks of treatment. WUE was then calculated as grams of shoot dry matter per liter of water used.

### 4.4. Statistical Analysis

Data from hydrotropic assay were subjected to a one–way analysis of variance (ANOVA) using SPSS software (version 26.0, IBM Corp., Armonk, NY, USA). The greenhouse FPRD experiment was analyzed by two–way analysis of variance (ANOVA) using the general linear model (univariate) procedure with SPSS. ANOVAs were performed for the effects of irrigation treatment, maize genotype, and their interactions. Multiple comparisons among maize varieties were conducted using Duncan’s test at a significant level of *p* ≤ 0.05. Differences between WW and FPRD conditions within the same maize genotype were conducted using a *t*-test. Results in text and figures are reported as mean ± standard error (SE). Pearson’s correlation coefficients were calculated among all trait variables under WW conditions and separately under FPRD conditions. A principal component analysis (PCA) was performed to visualize multivariate trait patterns. PCA was conducted on the correlation matrix of the measured shoot and root traits. Figures were prepared using Origin software (OriginLab, Northampton, MA, USA).

## Figures and Tables

**Table 1:** Summary of ANOVA for maize root dry weight, root water content, shoot dry weight, shoot water content, stem diameter, plant height, and water use efficiency (WUE) as influenced by irrigation treatment (IR) and genotype (G) in the greenhouse trial.

Sources	df	Root Dry Weight		Root Water Content		Shoot Dry Weight		Shoot Water Content		Stem Diameter		Plant Height		WUE	
(Factors)		*F* Value	*p* Value	*F* Value	*p* Value	*F* Value	*p* Value	*F* Value	*p* Value	*F* Value	*p* Value	*F* Value	*p* Value	*F* Value	*p* Value
IR	1	73.584	<0.001	20.668	<0.001	90.705	<0.001	107.645	<0.001	229.835	<0.001	157.545	<0.001	13.074	0.001
G	6	9.886	<0.001	2.310	0.051	0.742	0.619	11.028	<0.001	5.051	0.001	1.096	0.38	5.134	<0.001
IR×G	6	4.407	0.002	1.202	0.324	1.156	0.348	3.435	0.008	8.703	<0.001	0.457	0.836	3.286	0.01

## Data Availability

The data generated and analyzed during this study were fully presented in the main article and its Supplementary Files. Any additional raw data are available from the corresponding authors upon reasonable request.

## Supplementary Materials

The following supporting information can be downloaded at: https://www.mdpi.com/article/10.3390/plants15101571/s1. Figure S1. Phylogenetic analysis of maize varieties based on 5K chip. Data above the lines represented genetic distance. Table S1 The genetic background of the maize hybrids.

## Abbreviations

The following abbreviations are used in this manuscript:

PRD	Partial root–zone drying
WUE	Water use efficiency
PCA	Principal component analysis
PC1	The first principal component
PC2	The second principal component
FPRD	Fixed partial root–zone drying
WW	Well–watered
