# Supplementary material for: Genotypic Variation in Maize Root Hydrotropism and Its Association with Shoot Growth and Water Use Efficiency Under Partial Root–Zone Drying
Source: Plants (Basel). 2026 May 21;15(10):1571. doi: 10.3390/plants15101571 (PMC13210698; doi:10.3390/plants15101571)
Supplement: Supplementary file 1 [file plants-15-01571-s001.zip › Supplementary Figure S1.pdf]

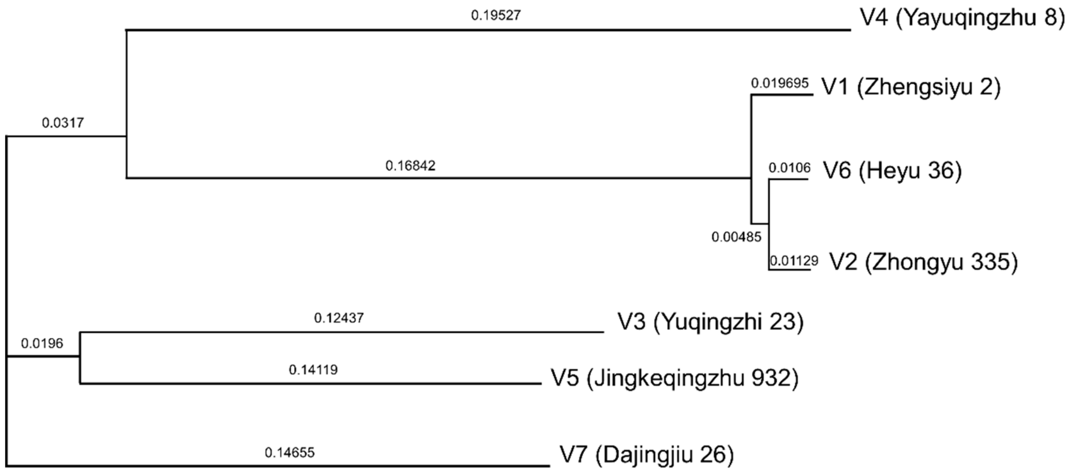

Figure S1. phylogenetic analysis of maize vatities based on 5K chip. Data above the lines represented genetic distance.
